# Gastrointestinal comorbidities associated with atrial fibrillation

**DOI:** 10.1186/2193-1801-3-603

**Published:** 2014-10-15

**Authors:** François Laliberté, Yuliya Moore, Katherine Dea, Joyce C LaMori, Samir H Mody, JaCinda L Jones, Michele D Arledge, C V Damaraju, Jeff R Schein, Patrick Lefebvre

**Affiliations:** Groupe d’analyse, Ltée, 1000 rue de la Gauchetière Ouest, Bureau 1200, Montréal, Québec H3B 4 W5 Canada; Janssen Scientific Affairs, LLC, Raritan, NJ USA

**Keywords:** Atrial fibrillation, Risk, Gastrointestinal conditions, Dyspepsia

## Abstract

This observational study was conducted to describe the risk of gastrointestinal (GI) events among patients with atrial fibrillation (AF). We analyzed Thomson Reuters MarketScan® data from 2005 to 2009. Subjects aged ≥18 years with ≥ 1 AF diagnosis were selected. GI events were identified from claims with a primary or secondary diagnosis code for any GI condition. The risk of GI events was assessed using cumulative incidence (new GI events/patients with AF without GI condition at baseline) and incidence rates (IRs), calculated as the number of patients with new GI events divided by patient-years of observation. In addition, the CHADS_2_ score was evaluated at baseline to determine the patient’s risk of stroke. A total of 557,123 AF patients were identified. The mean (median) AF patient age was 68.2 years (70); 45% were female. The cumulative incidences of any GI event and dyspepsia were 40% and 19%, respectively. The corresponding IRs were 38.8 and 14.7 events per 100 patient–years. IRs of any GI events for female and male patients were 43.6 and 35.5; for patients in the age groups <65, 65–74, 75–84, and ≥85 years, IRs were 32.3, 38.9, 44.6, and 52.7; for patients with a CHADS_2_ score of 0, 1–2, 3–4, and 5–6, IRs were 30.3, 41.6, 56.9, and 74.5, respectively. In this large claims database, 40% of AF patients experienced a GI event, predominantly dyspepsia. Physicians should take age and comorbidities into consideration when managing AF patients.

**Electronic supplementary material:**

The online version of this article (doi:10.1186/2193-1801-3-603) contains supplementary material, which is available to authorized users.

## Introduction

Atrial fibrillation (AF) is the most common clinical arrhythmia; an estimated 2.3 million Americans were suffering from this condition in 2010 (Fuster et al. 2001; Go et al. 2001). AF is also strongly age dependent, affecting approximately 11–12% of persons ≥80 years of age, compared with only 0.1–0.2% of persons ≤55 years of age (Go et al. 2001). AF is commonly associated with other cardiovascular diseases, hypertension, including congestive heart failure, valvular heart disease, and ischemic heart disease (Lloyd-Jones et al. 2004). However, while literature documenting cardiovascular comorbidities is plentiful, less attention has been given to the prevalence and impact of gastrointestinal (GI) conditions such as dyspepsia, gastroesophageal reflux disease (GERD), peptic ulcer diseases, and GI bleeding in patients with AF (Hernandez-Diaz & Rodriguez 2002; Locke et al. 1997; Talley et al. 1992; Talley et al. 1995). The GI tract has been documented as one of the most common locations of major bleeds attributed to a typical thromboprophylaxis regimen in stroke prevention (Coleman et al. 2012).

The number of GI conditions also increases with age (Hernandez-Diaz & Rodriguez 2002; Blachut et al. 2004; Garcia Rodriguez et al. 1998; Som et al. 2010; Sostres et al. 2010). Dyspepsia, for example, is a common condition in the elderly. It is also a likely comorbidity in patients with AF. In a recent retrospective observational study, subjects with AF presenting with dyspepsia tended to have a greater health burden and lower quality of life than those without dyspepsia. Moreover, these patients were at greater risk of stroke (Lamori et al. 2012). The agents used in patients with AF to prevent stroke or treat other comorbidities are known to increase the risk of GI events. These agents include, but are not limited to, anticoagulants, nonsteroidal anti-inflammatory drugs (NSAIDs) (e.g. aspirin), corticosteroids, and calcium channel blockers (Garcia Rodriguez et al. 1998; Bytzer 2010). Agents currently used to treat patients with GI conditions or to counteract treatment-induced GI events typically include acid secretory inhibitors, such as proton pump inhibitors (PPIs) (Bytzer 2010; McGowan et al. 2008; Yeomans et al. 1998).

GI conditions, in particular GERD, also have been proposed as a potential independent trigger for AF, because of the close anatomical positioning of the esophagus and the atria, and their similar nerve innervations (i.e. vagal nerve innervation) (Huang et al. 2012). The fact that vagal nerve overstimulation has been observed in patients with GERD and has been suggested as a contributing factor in AF supports the notion of GERD-mediated AF stimulation via vagal innervation. The most compelling evidence in support of GERD-mediated AF stimulation was found in a recent nationwide population-based survey in Taiwan, where GERD was reported to be independently associated with an increased risk of developing concomitant AF (Huang et al. 2012). It is thought that the prevalence of GERD increases with age. Whether this is the case has not yet been fully elucidated; nevertheless, esophageal symptoms (i.e. severe reflux esophagitis) have been reported to be more severe in older patients (Becher & El Serag 2011).

In the US, the management of AF is dictated by guidelines issued by the American College of Chest Physicians, which use the CHADS_2_ classification to estimate stroke risk. This is established by adding points relating to risk factors of stroke – **C**ongestive heart failure, **H**ypertension, **A**ge ≥75 years, **D**iabetes mellitus, prior **S**troke or transient ischemic attack or thromboembolism: a higher score denotes a greater risk (Gage et al. 2004; Singer et al. 2008). Oral anticoagulant therapy is recommended for patients with a CHADS_2_ score ≥2, while either warfarin or aspirin is recommended for patients with a CHADS_2_ score of 1 (Singer et al. 2008).

In light of the prevalence of GI comorbidities in patients with AF and to better understand how it affects this population, we conducted an observational study to document the extent of GI comorbidities in patients diagnosed with AF.

## Methods

### Data source

Health insurance claims from the Thomson Reuters MarketScan® database were used to conduct the analysis. The MarketScan database, which combines two separate databases (Commercial Claims and Encounters and Medicare Supplemental and Coordination of Benefits [COB]) to cover all age groups, contains claims from ~100 employers, health plans, and government and public organizations representing about 30 million covered lives. All US census regions are represented, with the South and North Central (Midwest) regions predominating. The MarketScan data used in the current analysis covered the period from January 2005 through December 2009.

Data used in the present study included health plan enrollment records, patient demographics, inpatient and outpatient medical services, and outpatient prescription drug dispensing records. Data included in the MarketScan database are de-identified and are in compliance with the Health Insurance Portability and Accountability Act (HIPAA) of 1996 to preserve patient anonymity and confidentiality.

### Study design

A retrospective longitudinal cohort design was employed. To be included in the study sample, patients were required to meet the following criteria: (i) have at least one primary or secondary diagnosis of AF (ICD-9-CM code 427.31), (ii) have continuous health plan enrollment during the study period, and (iii) be at least 18 years of age as of the date of the index AF (index date). In addition, patients were required to have continuous health plan enrollment for at least 180 days prior to the index date (baseline/washout period). The observation period of patients spanned from the index date through the earlier of either the health plan disenrollment date or the end of data availability.

### Outcome measures

The main endpoint of the study included the risk of GI events. These were defined as a primary or secondary diagnosis code for any GI event (see Additional file 1 for a complete list of ICD-9-CM codes) and the subset of GI events based on the classification in the recent Randomized Evaluation of Long-Term Anticoagulation (RE-LY) study (Connolly et al. 2009), including dyspepsia (including upper abdominal pain, abdominal pain, and abdominal discomfort, as well as dyspepsia), diarrhea, vomiting, and GI bleeding.

The secondary endpoints of the study included the following GI conditions: constipation, intestinal diverticula, dysphagia, esophagitis, flatulence, eructation and gas pain, gastritis and duodenitis, GERD, malignant neoplasm of the digestive organs and peritoneum, nausea alone, non-infectious gastroenteritis and colitis, other disorders of the intestine, and peptic ulcer diseases. GI-related hospitalization was also reported; this was defined as a hospitalization that had any GI-related ICD-9-CM code associated with it, either as a primary or secondary diagnosis.

Rates of GI events were also calculated among subgroups with respect to gender, age (<65 years, 65–74 years, 75–84 years, and ≥85), and CHADS_2_ score (0, 1–2, 3–4, and 5–6). The CHADS_2_ score consisted of the following stroke risk factors evaluated at baseline: congestive heart failure, hypertension, age ≥75, diabetes, and prior stroke or transient ischemic attack (2 points for prior stroke or transient ischemic attack and 1 point each for other factors) (Singer et al. 2008).

### Statistical analyses

Descriptive statistics were used to describe patient baseline characteristics. Means and standard deviations (SDs) were used to describe continuous variables; frequencies and percentages were reported for categorical variables.

The prevalence of GI events was calculated as the number of patients with a GI event during the 180-day baseline and/or study follow-up period divided by the total number of AF patients. Cumulative incidence, calculated as the number of patients with a new GI event (i.e. post-index AF diagnosis only) divided by the total number of AF patients without a history of GI events at baseline, was also reported. The 95% confidence intervals (CIs) of the prevalence and cumulative incidence of GI events were computed using binomial distribution.

Finally, the incidence rates (IRs) of GI events were calculated as the number of new GI cases divided by patient-years of observation, which was censored at the time of the first event. This person-time approach is used to account for different lengths of observation among study subjects in a non–experimental setting. IR was expressed as number of new cases per 100 patients per year. The 95% CIs of the IRs of GI events were computed using the Poisson distribution. All statistical analyses were performed using SAS version 9.2 (SAS Institute, Inc., Cary, NC).

## Results

### Patient characteristics

A total of 557,123 patients with AF met the inclusion criteria and formed the study population, of whom 143,955 (25.8%) had a history of GI conditions during the 180-day baseline period. Table 1 describes the baseline characteristics of the study population. The mean age (median; SD) was 68.2 (70; 14.9) years, and 249,331 patients (44.8%) were female. CHADS_2_ scores at baseline were 0, 1–2, 3–4, and 5–6, for 165,936 (29.8%), 318,530 (57.2%), 66,436 (11.9%), and 6,221 (1.1%) patients, respectively. Comorbidities at baseline included cardiovascular diseases (53.1%), hypertension (39.3%), diabetes (20.0%), cancer (13.5%), arthritis (12.7%), and chronic kidney disease (6.5%).

**Table 1:** **Patient characteristics of the AF study population**

Variable	All AF patients (N = 557,123)	AF patients without history of GI at baseline (N = 413,168)
Mean follow-up period, days (SD)	543.2 (455.0)	563.3 (457.9)
Demographics		
Age, years, mean (SD) [median]	68.2 (14.9) [70]	67.7 (15) [69]
Female, n (%)	249,331 (44.8%)	177,888 (43.1%)
Region, n (%)		
South	210,204 (37.7%)	155,089 (37.5%)
West	93,857 (16.8%)	71,646 (17.3%)
North Central	179,174 (32.2%)	131,464 (31.8%)
Northeast	72,331 (13.0%)	53,792 (13.0%)
Unknown	1,557 (0.3%)	1,177 (0.3%)
Type of insurance, n (%)		
Comprehensive	177,702 (31.9%)	129,040 (31.2%)
EPO	1,335 (0.2%)	1,076 (0.3%)
HMO	69,097 (12.4%)	53,120 (12.9%)
POS	26,145 (4.7%)	19,932 (4.8%)
PPO	259,366 (46.6%)	192,305 (46.5%)
POS with capitation	2,308 (0.4%)	1,808 (0.4%)
CDHP	4,567 (0.8%)	3,689 (0.9%)
Other/Unknown	16,603 (3.0%)	12,198 (3.0%)
Year of index date, n (%)		
2005	81,555 (14.6%)	62,282 (15.1%)
2006	105,630 (19.0%)	79,372 (19.2%)
2007	108,170 (19.4%)	80,423 (19.5%)
2008	123,788 (22.2%)	91,663 (22.2%)
2009	137,980 (24.8%)	99,428 (24.1%)
Charlson comorbidity index^a^, mean (SD)	1.36 (1.93)	1.05 (1.57)
CHADS_2_^a,b^ score, n (%)		
0	165,936 (29.8%)	134,997 (32.7%)
1-2	318,530 (57.2%)	233,956 (56.6%)
3-4	66,436 (11.9%)	41,209 (10.0%)
5-6	6,221 (1.1%)	3,006 (0.7%)
History of GI^a^, n (%)		
Any GI condition	143,955 (25.8%)	0 (0.0%)
GI events based on the RE-LY study classification^c^	87,017 (15.6%)	0 (0.0%)
Dyspepsia^d^	64,202 (11.5%)	0 (0.0%)
Diarrhea	12,693 (2.3%)	0 (0.0%)
Vomiting	15,448 (2.8%)	0 (0.0%)
Gastrointestinal bleeding	15,466 (2.8%)	0 (0.0%)
Other GI events		
Gastroesophageal reflux disease	21,373 (3.8%)	0 (0.0%)
Diverticula of intestine	19,640 (3.5%)	0 (0.0%)
Other disorders of intestine	15,008 (2.7%)	0 (0.0%)
Gastritis and duodenitis	13,923 (2.5%)	0 (0.0%)
Dysphagia	13,306 (2.4%)	0 (0.0%)
Constipation	10,669 (1.9%)	0 (0.0%)
Noninfectious gastroenteritis and colitis	9,166 (1.6%)	0 (0.0%)
Esophagitis	9,220 (1.7%)	0 (0.0%)
Nausea Alone	6,532 (1.2%)	0 (0.0%)
Malignant neoplasm of digestive organs and peritoneum	9,702 (1.7%)	0 (0.0%)
Flatulence, eructation, and gas pain	4,868 (0.9%)	0 (0.0%)
Peptic ulcer diseases	4,352 (0.8%)	0 (0.0%)
Any GI-related hospitalization	43,851 (7.9%)	0 (0.0%)
Other comorbidities^a^, n (%)		
Cardiovascular diseases	295,617 (53.1%)	202,017 (48.9%)
Chronic kidney disease	36,187 (6.5%)	20,521 (5.0%)
Diabetes	111,423 (20.0%)	76,751 (18.6%)
Hypertension	218,765 (39.3%)	148,707 (36.0%)
Arthritis	70,882 (12.7%)	47,053 (11.4%)
Any cancer	74,954 (13.5%)	42,829 (10.4%)
Baseline medication^a^, n (%)		
Medications that may cause GI events	359,398 (64.5%)	257,357 (62.3%)
Anticoagulants	105,367 (18.9%)	80,059 (19.4%)
Antiplatelets	55,143 (9.9%)	37,887 (9.2%)
Corticosteroids	58,065 (10.4%)	36,247 (8.8%)
NSAIDs	67,179 (12.1%)	47,315 (11.5%)
SSRIs	57,567 (10.3%)	37,959 (9.2%)
Calcium channel blockers	107,587 (19.3%)	77,563 (18.8%)
Bisphosphonates	35,491 (6.4%)	24,934 (6.0%)
Antibiotic	154,004 (27.6%)	105,071 (25.4%)
Pain medications (opioids)	134,460 (24.1%)	84,629 (20.5%)
Antineoplastic	15,634 (2.8%)	8,978 (2.2%)
Anesthesia medication	236 (0.0%)	119 (0.0%)
Medications used to treat poisonings	145 (0.0%)	80 (0.0%)
Iron-related medication	89 (0.0%)	44 (0.0%)
Medications used to treat GI events	162,016 (29.1%)	91,083 (22.0%)
Antacids	124 (0.0%)	78 (0.0%)
Antidiarrheals	6,895 (1.2%)	2,592 (0.6%)
Antiemetics	14,165 (2.5%)	7,138 (1.7%)
Digestive aids	1,198 (0.2%)	429 (0.1%)
Gastrointestinal medications	19,550 (3.5%)	7,709 (1.9%)
Laxatives	24,122 (4.3%)	7,893 (1.9%)
Ulcer drugs	133,053 (23.9%)	76,924 (18.6%)
Proton Pump Inhibitors	110,762 (19.9%)	62,566 (15.1%)
H-2 Antagonists	22,720 (4.1%)	14,862 (3.6%)

*AF* atrial fibrillation, *GI* gastrointestinal, *EPO* Exclusive Provider Organization, *HMO* health maintenance organization, *POS* point of service, *PPO* preferred provider organization, *CDHP* Consumer Directed Health Plans, *SSRI* selective serotonin reuptake inhibitors, *NSAIDs* nonsteroidal anti-inflammatory drugs.

^a^Based on a baseline period of 180 days prior to index date.

^b^CHADS_2_ score was calculated as 1 point for congestive heart failure, hypertension, age ≥75, and diabetes mellitus, and 2 points for prior stroke or transient ischemic attack (Source: Gage Circulation 2004).

^c^Including dyspepsia, diarrhea, vomiting, and gastrointestinal bleeding.

^d^Including abdominal pain upper, abdominal pain, abdominal discomfort, and dyspepsia.

Medications that may cause GI events were taken by 359,398 (64.5%) patients with AF at baseline, the most frequent (>10%) drug classes being antibiotics (27.6%), opioid pain medications (24.1%), calcium channel blockers (19.3%), anticoagulants (18.9%), non-steroidal anti-inflammatory drugs (NSAIDs; 12.1%), selective serotonin reuptake inhibitors (SSRIs; 10.3%), and corticosteroids (10.4%).

Medications used to treat GI events were taken by 162,016 (29.1%) patients with AF, among whom 110,762 (19.9%) used PPIs, 24,122 used laxatives (4.3%), 22,720 used H-2 antagonists (4.1%), 19,550 used gastrointestinal medications (3.5%), 14,165 used antiemetics (2.5%), 6,895 used antidiarrheals (1.2%), 1,198 used digestive aids (0.2%), and 124 used antacids (0.0%).

### Treatment patterns

Table 2 presents the treatment patterns of medications associated with GI conditions during the observation period. The mean (±SD) observation period for patients with AF was 543 ± 455 days (Table 1). During the follow-up, 398,633 (71.6%) patients took at least one medication that may cause GI events: anticoagulant and antiplatelet agents were taken by 37.5% and 12.0% of patients with AF, respectively, whereas 225,833 (40.5%) patients took at least one medication used to treat GI events (Table 2).The mean (±SD) exposures to medications that may cause GI events and to medications used to treat GI events were 524 ± 453 and 393 ± 410 days, respectively (Table 2).

**Table 2:** **Treatment patterns of medications associated with GI conditions**

Variable	All AF patients (N = 557,123)	AF patients without history of GI at baseline (N = 413,168)
Medications associated with GI, n (%)		
Medications that may cause GI events^a^	398,633 (71.6%)	298,490 (72.2%)
Medications used to treat GI events^b^	225,833 (40.5%)	154,164 (37.3%)
Exposure to therapy^c^, days, mean (SD)		
Medications that may cause GI events^a^	523.7 (452.7)	534.8 (456.5)
Medications used to treat GI events^b^	392.6 (409.6)	383.1 (408.6)
Dispensings per patient, mean (SD)		
Medications that may cause GI events^a^	17.6 (21.2)	17.4 (20.8)
Medications used to treat GI events^b^	7.4 (9.3)	7.0 (8.8)
Days of supply per dispensing per patient, mean (SD)		
Medications that may cause GI events^a^	34.7 (22.3)	35.5 (22.8)
Medications used to treat GI events^b^	43.4 (28.6)	43.0 (29.2)
Anticoagulant and antiplatelet agents use, n (%)		
Antiplatelet	66,790 (12.0%)	48,808 (11.8%)
Anticoagulant	208,985 (37.5%)	162,597 (39.4%)

*AF* atrial fibrillation, *GI* gastrointestinal.

^a^Including anticoagulant, antiplatelet, corticosteroids, NSAIDs, SSRIs, calcium channel blockers, bisphosponates, antibiotic, pain medications (opioids), antineoplastic, anesthesia medication, medications used to treat poisonings, and iron-related medication.

^b^Including antacids, antidiarrheals, antiemetics, digestive aids, gastrointestinal agents, laxatives, and ulcer drugs.

^c^Time from the date of the first dispensing to the end of the days of supply for the last dispensing.

### Risk of GI events

Table 3 presents the prevalence and cumulative incidence of GI events. Over the 180-day baseline and mean follow-up of 543 days, 308,823 (55.4%) patients had at least one GI event, 215,942 (38.8%) had at least one GI event based on the RE-LY study classification, and 121,189 patients (21.8%) had at least one GI-related hospitalization. Dyspepsia was the most common GI event, occurring in 29.6% of AF patients. The other most frequent GI events (≥5%) included intestinal diverticula (n = 62,638; 11.2%), GERD (n = 63,159; 11.3%), GI bleeding (n = 52,979; 9.5%), other disorders of the intestine (n = 49,736; 8.9%), vomiting (n = 46,866, 8.4%), gastritis and duodenitis (n = 46,974; 8.4%), dysphagia (n = 46,506; 8.3%), diarrhea (n = 43,628; 7.8%), constipation (n = 35,832; 6.4%), non-infectious gastroenteritis and colitis (n = 29,602; 5.3%), and esophagitis (n = 28,092; 5.0%).

**Table 3:** **Prevalence and cumulative incidence of GI events**

Variable	All patients with AF (N = 557,123)		AF patients without history of GI at baseline (N = 413,168)	
	Number of GI cases ^a^	Prevalence of GI (per 100 persons) [95% CI ^b^]	Number of incident GI cases ^c^	Cumulative incidence (per 100 persons) [95% CI ^b^]
Any GI event	308,823	55.4 [55.3,55.6]	164,868	39.9 [39.8,40.1]
GI events based on the RE-LY study classification^d^	215,942	38.8 [38.6,38.9]	108564	26.3 [26.1,26.4]
Dyspepsia^e^	164,892	29.6 [29.5,29.7]	79,069	19.1 [19.0,19.3]
Gastrointestinal bleeding	52,979	9.5 [9.4,9.6]	26,067	6.3 [6.2,6.4]
Vomiting	46,866	8.4 [8.3,8.5]	20,297	4.9 [4.8,5.0]
Diarrhea	43,628	7.8 [7.8,7.9]	19,928	4.8 [4.8,4.9]
Other GI events				
Diverticula of intestine	62,638	11.2 [11.2,11.3]	30,983	7.5 [7.4,7.6]
Gastroesophageal reflux disease	63,159	11.3 [11.3,11.4]	28,938	7.0 [6.9,7.1]
Other disorders of intestine	49,736	8.9 [8.9,9.0]	23,435	5.7 [5.6,5.7]
Dysphagia	46,506	8.3 [8.3,8.4]	22,071	5.3 [5.3,5.4]
Gastritis and duodenitis	46,974	8.4 [8.4,8.5]	21,422	5.2 [5.1,5.3]
Constipation	35,832	6.4 [6.4,6.5]	16,729	4.0 [4.0,4.1]
Noninfectious gastroenteritis and colitis	29,602	5.3 [5.3,5.4]	12,837	3.1 [3.1,3.2]
Esophagitis	28,092	5.0 [5.0,5.1]	11,968	2.9 [2.8,2.9]
Nausea Alone	22,673	4.1 [4.0,4.1]	9,962	2.4 [2.4,2.5]
Malignant neoplasm of digestive organs and peritoneum	18,107	3.3 [3.2,3.3]	5,534	1.3 [1.3,1.4]
Flatulence, eructation and gas pain	17,263	3.1 [3.1,3.1]	7,209	1.7 [1.7,1.8]
Peptic ulcer diseases	13,914	2.5 [2.5,2.5]	6,311	1.5 [1.5,1.6]
Any GI-related hospitalization	121,189	21.8 [21.6,21.9]	55,744	13.5 [13.4,13.6]

*AF* atrial fibrillation, *GI* gastrointestinal.

^a^Including GI events observed during the 180-day baseline or study follow-up period.

^b^The 95% confidence intervals of GI events were computed using the binomial distribution.

^c^Including GI events observed only during the study follow-up period (i.e., patients with history of GI at baseline were excluded).

^d^Including dyspepsia, diarrhea, vomiting, and gastrointestinal bleeding.

^e^Defined as abdominal pain upper, abdominal pain, abdominal discomfort, and dyspepsia.

### AF patients without a history of GI conditions at baseline

**Figure 1:**
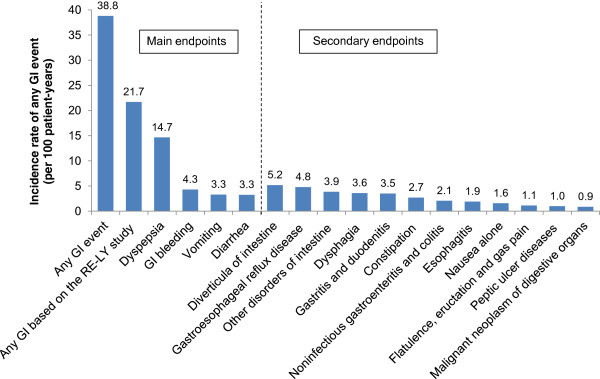
**Incidence rate of any gastrointestinal (GI) events in atrial fibrillation patients without history of GI at baseline (N = 413,168).**

**Figure 2:**
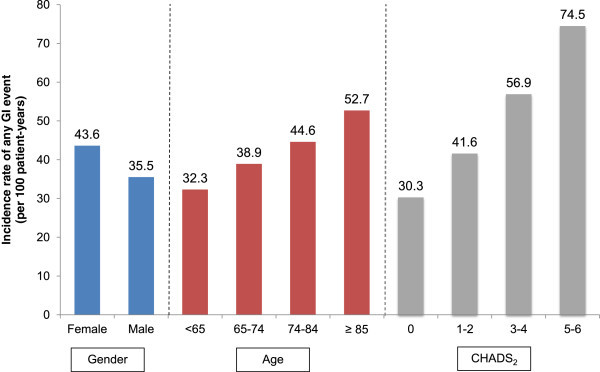
**Incidence rate of any gastrointestinal (GI) events stratified by gender, age, and CHADS**_**2**_ **(Congestive heart failure, Hypertension, Age ≥75 years, Diabetes mellitus, prior Stroke or transient ischemic attack or thromboembolism) (N = 413,168).**

For AF patients without a history of GI conditions at baseline, the cumulative incidences of any GI event, any GI event based on the RE-LY study classification, and dyspepsia were 39.9%, 26.3%, and 19.1%, respectively. The corresponding IRs were 38.8, 21.7, and 14.7 events per 100 patient–years, respectively (Figure 1). The IRs of any GI event for female and male patients were 43.6 and 35.5, respectively (Figure 2). The IRs of any GI event increased with age and CHADS_2_ score: for patients in the age groups <65, 65–74, 75–84, and ≥85 years, IRs were 32.3, 38.9, 44.6, and 52.7, respectively; for patients with a CHADS_2_ score of 0, 1–2, 3–4, and 5–6, IRs were 30.3, 41.6, 56.9, and 74.5, respectively.

## Discussion

Our analysis of real-world data demonstrates that a large proportion of patients with AF are at high risk of GI events. GI events were observed in more than half of the study population, with a prevalence of 55.4 per 100 persons. Dyspepsia was the most common GI symptom, reported in 557,123 patients with AF (29.6%; 164,892/557,123), accounting for 54% of all 308,823 GI events reported. Dyspepsia is regarded as a significant burden for AF patients (Lamori et al. 2012), and in several studies of patients treated with NSAIDs and aspirin, an important reason for discontinuing treatment (CAPRIE Steering Committee 1996; Cryer et al. 2011; Niculescu et al. 2009; Ofman et al. 2003; Peto et al. 1988; Saini et al. 2009; Tournoij et al. 2009). Other common GI effects included intestinal diverticula (62,638), GERD (63,159), and GI bleeding (52,979). GERD was recently found to be both a trigger for AF and associated with its development (Huang et al. 2012).

Consistent with past studies of cardiovascular disorders associated with AF (Carroll & Majeed 2001), our study found cardiovascular diseases and hypertension to be frequent at baseline. Medications that can elicit GI adverse effects, ranging from dyspepsia to GI bleeding, include aspirin, other antiplatelet medications, anticoagulants, antibiotics, corticosteroids, SSRIs, NSAIDs, bisphosphonates, opioids and pain medications, calcium channel blockers, and iron-related medications, which are used to treat cardiovascular disorders and other comorbidities (e.g. depression and arthritis) (Garcia Rodriguez et al. 1998; Sostres et al. 2010; Bytzer 2010; Ashberg et al. 2010; Diego et al. 2011; Gabriel et al. 1991). In the current study, the most commonly used medications reported at baseline in AF patients, with known GI adverse effects, included pain medications (opioids), antibiotics, calcium channel blockers, and anticoagulants; 359,398 patients (64.5%) received at least one medication that may cause GI events, and that proportion rose to 71.6% after the index diagnosis of AF. This finding could partly explain why, over the entire study period, 55.4% of patients with AF had at least one GI event. Although a large proportion of patients presented with GI events in our study, only 40.5% of patients with AF received treatment to counteract these events compared to 29.1% at baseline, where 80% of patients treated used at least one ulcer drug (i.e., PPIs or H-2 antagonists).

In our study, we assumed that a number of patients with AF would have received more than one medication that could cause a GI event. The use of multiple medications by older patients reflects the multiple comorbidities in this population (Hajjar et al. 2007) and can substantially increase their risk for GI events. For example, SSRIs increase the risk of GI bleeding up to three times and, when used concomitantly with NSAIDs, up to 15 times (Ashberg et al. 2010). Warfarin used concomitantly with aspirin, anti-infective agents, or NSAIDs, also has been shown to increase the risk of GI bleeding (Ashberg et al. 2010; Hallas et al. 2006; Man-Son-Hing & Laupacis 2003; Schelleman et al. 2008; Shorr et al. 1993). Moreover, dual therapy in thromboprophylaxis has been found to increase patients’ odds of experiencing a major GI bleed compared with monotherapy. Administering the antiplatelet agent clopidogrel with aspirin increased patients’ odds of having a major GI bleed by 93% compared with aspirin monotherapy (Coleman et al. 2012). Given the greater risk for stroke with older age, we may assume that patients in this age group are more likely to be candidates for dual thromboprophylaxis therapy and are therefore at greater risk for the subsequent GI effects attributed to this regimen.

Consistent with previous findings, in our study, advancing age was found to increase the risk of GI conditions, ranging from IRs of any GI event of 32.3 per 100 patient-years for patients aged <65 years to corresponding IRs of 52.7 per 100 patient-years for patients aged ≥85 years. A higher CHADS_2_ score, indicative of greater comorbidity, also was associated with a higher risk of GI conditions, ranging from IRs of 30.3 per 100 patient-years for a CHADS_2_ score of 0 to corresponding IRs of 74.5 per 100 patient-years for a CHADS_2_ score of 5–6. Notably, subjects with higher CHADS_2_ scores tend to be older (i.e. ≥75 years) (Oldgren et al. 2011). Given that the risk of GI events increases with age and that AF is strongly age dependent, this study highlights the importance of profiling the characteristics of patients with AF, in terms of both comorbidities and age, when making treatment decisions. We suggest that further research on GI adverse events in AF patients, specifically regarding the potential impact of AF therapy and age on GI conditions, is warranted. Moreover, the propensity for GI conditions, such as GERD, to trigger AF requires further elucidation. The possible impact of GI events and other comorbidities on the underuse of anticoagulants in AF patients also might be explored in future research.

Our study has a number of limitations. First, claims databases may contain inaccuracies or omissions in coded procedures, diagnoses, or pharmacy claims; however, it would be unlikely that these have significantly impacted our results considering the large sample size and the relatively high proportion of patients having a GI event in our study. Second, antiplatelet therapy was assessed based on pharmacy dispensing claims, and because the data do not capture nonprescription medications, such as aspirin, we may have underestimated antiplatelet utilization. Third, some medications used to treat GI conditions are also available without a prescription, which may further underestimate the utilization of these agents. In addition, the observational design was susceptible to various biases, such as information or classification bias (e.g. the identification of false positives of GI events). Despite these limitations, well-designed observational studies provide valuable information, with real-life scenarios and high generalizability.

## Conclusion

This large population-based study of more than 500,000 patients, based on real-world data, indicates a high risk of GI events, predominantly dyspepsia, among patients with AF. In view of the fact that GI comorbidities commonly coexist with AF, particularly in the elderly, it is important to take these comorbidities into account when managing AF.

## Electronic supplementary material

Additional file 1: **ICD-9-CM diagnosis codes used to identify gastrointestinal conditions.** (DOC 84 KB)
