# Supplementary material for: Gastrointestinal comorbidities associated with atrial fibrillation
Source: Springerplus. 2014 Oct 15;3:603. doi: 10.1186/2193-1801-3-603 (PMC4210454; doi:10.1186/2193-1801-3-603)
Supplement: Supplementary file 1 — Additional file 1: ICD-9-CM diagnosis codes used to identify gastrointestinal conditions. (DOC 84 KB) [file 40064_2014_1315_MOESM1_ESM.doc]

**Additional file 1.** ICD-9-CM diagnosis codes used to identify gastrointestinal conditions

| Medical Term | Code(s) | Description |
| --- | --- | --- |
| Dyspepsia | 536.8 | Dyspepsia disorders of function of stomach |
|  | 789.0x | Abdominal pain |
| Diarrhea | 787.91 | Diarrhea |
| Vomiting | 536.2 | Persistent vomiting |
|  | 643.x | Excessive vomiting in pregnancy |
|  | 787.0 | Nausea and vomiting |
|  | 787.01 | Nausea with vomiting |
|  | 787.03 | Vomiting alone |
| Gastrointestinal bleeding | 578.0 | Hematemesis |
|  | 578.1 | Blood in stool |
|  | 578.9 | Hemorrhage of gastrointestinal tract unspecified |
| Constipation | 564.0x | Functional digestive disorders not elsewhere classified |
| Diverticula of intestine | 562.0 | Diverticula of small intestine |
|  | 562.1 | Diverticula of colon |
| Dysphagia | 787.2x | Dysphagia |
| Esophagitis | 530.1.x | Esophagitis |
| Flatulence, eructation, and gas pain | 787.3 | Flatulence eructation and gas pain |
| Gastritis and duodenitis | 535.0x | Acute gastritis |
|  | 535.1x | Atrophic gastritis |
|  | 535.2x | Gastric mucosal hypertrophy |
|  | 535.3x | Alcoholic gastritis |
|  | 535.4x | Other specified gastritis |
|  | 535.5x | Unspecified gastritis and gastroduodenitis |
|  | 535.6x | Duodenitis |
|  | 535.7x | Eosinophilic gastritis |
| Gastroesophageal reflux disease | 530.81 | Esophageal reflux (GERD) |
|  | 787.1 | Heartburn |
| Malignant neoplasm of digestive organs and peritoneum | 150.x | Esophagus |
|  | 151.x | Stomach |
|  | 152.x | Small intestine |
|  | 153.x | Colon |
|  | 154.x | Rectum, rectosigmoid junction, and anus |
|  | 155.x | Liver and intrahepatic bile ducts |
|  | 156.x | Gallbladder and extrahepatic bile ducts |
|  | 157.x | Pancreas |
|  | 158.x | Retroperitoneum and peritoneum |
|  | 159.x | Other and ill-defined sites |
| Nausea alone | 787.02 | Nausea alone |
| Noninfectious gastroenteritis and colitis | 558.1 | Gastroenteritis and colitis due to radiation |
|  | 558.2 | Toxic gastroenteritis and colitis |
|  | 558.3 | Allergic gastroenteritis and colitis |
|  | 558.4x | Eosinophilic gastroenteritis and colitis |
|  | 558.9 | Other and unspecified |
| Other disorders of intestine | 569.0 | Anal and rectal polyp |
|  | 569.1 | Rectal prolapse |
|  | 569.2 | Stenosis of rectum and anus |
|  | 569.3 | Hemorrhage of rectum and anus |
|  | 569.4x | Other specified disorders of rectum and anus |
|  | 569.5 | Abscess of intestine |
|  | 569.6x | Colostomy and enterostomy complications |
|  |  |  |
|  | 569.7x | Complications of intestinal pouch |
|  | 569.8x | Other specified disorders of intestine |
|  | 569.9 | Unspecified disorder of intestine |
| Peptic ulcer diseases | 531.x | Gastric ulcer |
|  | 532.x | Duodenal ulcer |
|  | 533.x | Peptic ulcer site unspecified |
|  | 534.x | Gastrojejunal ulcer |
| Others GI events | 251.5 | Abnormality of secretion of gastrin |
|  | 307.53 | Rumination disorder |
|  | 536.3 | Gastroparesis |
|  | 530.x | Diseases of esophagus |
|  | 536.0 | Achlorhydria |
|  | 536.1 | Acute dilatation of stomach |
|  | 536.4x | Gastrostomy complications |
|  | 536.9 | Unspecified functional disorder of stomach |
|  | 537.x | Other disorders of stomach and duodenum |
|  | 538.x | Gastrointestinal mucositis (ulcerative) |
|  | 787.4 | Visible peristalsis |
|  | 787.5 | Abnormal bowel sounds |
|  | 787.6 | Incontinence of feces |
|  | 787.7 | Abnormal feces |
|  | 787.9x | Other symptoms involving digestive system |
|  | 564.x | Functional digestive disorders not elsewhere classified |
